# Passing Events and Topics

**Published:** 1921-01-22

**Authors:** 


### Passing Events and Topics.

### Sanitary Congress.

The Right Hon. the Earl of Radnor has consented to accept the office of President of the thirty-second Congress of the Royal Sanitary Institute, to be held at Folkestone from June 20 to 25, 1921.

### An Infirmary's Future.

The future of the Shrewsbury Workhouse Infirmary is uncertain. The buildings, used as a military hospital during the war, with accommodation for five hundred inmates, have been under offer to the County Council as a hospital for mental defectives. The Mental Deficiency Committee of the County Council states, however, that it is unlikely, in view of the Government's request to local authorities to incur no new expenditure, that the County Council will purchase the building at the price, viz., £20,000. As this is but the valuation price, the Board of Guardians is stated to prefer to make an effort to adapt the buildings as a hospital for the sick poor of Shrewsbury Union.

### First Entertainment for Seven Years.

The St. Bartholomew's Hospital Amateur Dramatic Club gave an entertainment to the medical, nursing, and working staffs of the hospital and their friends on "Twelfth Night," January 6. The performance, which took place in the Great Hall, was spirited and bright throughout. This is the first such entertainment given by this Club for seven years.

### Presentation to Aberavon Hospital.

The directors and workmen of the Port Talbot Steel Co. have commemorated the twenty-nine men from the steel-works who fell in the war by presenting to the Aberavon Hospital an electrical equipment, valued at £1,500, which they have also undertaken to maintain free of cost. In the entrance hall of the hospital a roll of honour in bronze has been affixed. Mrs. J. M. Turnock, wife of one of the directors, presented medals to the members of the nursing staff as a memento of the occasion.

### Entertainment at St. Mary's.

On Thursday, January 13, at the St. Mary's Hospital Musical and Dramatic Society's meeting the various troupes who performed in the wards on Christmas Day gave an entertainment for the benefit of the staff and students who were unable to be present at Christmas. The Society, which has now been in being one year, must be congratulated on the high standard attained by its members. The programme included songs by the pierrot troupe; the Jolly Idlers enacted a melodrama entitled "The Mystery of Lady Humphrietta's Pearls," by a cast of "cinema stars" among the students, and a modernised version of "Cinderella," in which Prince Bountiful, suffering from an obscure blood disease, was restored to life and health by a blood transfusion.

### A Strike and a Baby.

In order to provide the electric power to heat the incubator at the Ilford Maternity Home, where a nine weeks' old baby, weighing only 2¾ lb. at birth, is being reared, four electricians, working in six-hour shifts, have volunteered to remain at their post to work the necessary plant during the strike of the municipal employees.

### A Novel Appeal.

A motor-car, with a large clock on the front of the "bonnet," emphasising the fact that it takes £2 to support the London Hospital for five minutes, is paying a visit to every publican in London for the purpose of collecting funds.

### Hospital Saturday Fund's New Chairman.

The Right Hon. the Earl of Malmesbury has been elected Chairman of the Hospital Saturday Fund, London.

### Lectures on Physics.

Four lectures on "Communicable Diseases" will be delivered by Sir Robert Armstrong-Jones at Gresham College, Basinghall Street, E.C., on January 25, 26, 27, and 28, at 6 p.m. Admission will be free to men and women.

